# Investigation on the association between college students’ smartphone-related behaviors and sleep quality during COVID-19

**DOI:** 10.1371/journal.pone.0321060

**Published:** 2025-04-29

**Authors:** Qi Chen, Nur Syuhada binti Mat Sin, Afeez Nawfal bin Mohd Isa, Duobao Chen

**Affiliations:** 1 Faculty of Art, Sustainability & Creative Industry, Sultan Idris Education University, Tanjong Malim, Malaysia; 2 School of Medicine, Anhui University of Science and Technology, Huainan, China; Charité - Universitätsmedizin Berlin, GERMANY

## Abstract

**Objective:**

This study examines the quantifiable effects of pre-sleep smartphone use on sleep quality among college students during the COVID-19 pandemic, aiming to identify high-risk behaviors and inform targeted interventions.

**Methods:**

Based on data from 508 students of different genders and academic years, the study first conducted univariate and multivariate logistic regression analyses to explore the association between smartphone-related behaviors and sleep quality. Subsequently, the adjusted smartphone behaviors were stratified by gender(male/female) and academic year(freshmen/Sophomore/juniors) into subsets for further subgroup analysis, aiming to examine the relationship and impact of smartphone-related behaviors on sleep disturbancess across different genders and academic years.

**Results:**

he analysis revealed that specific pre-sleep activities were significantly associated with sleep quality. Notably, playing games before sleep (OR=6.071, p<0.001), late-night phone use (OR=2.824, p=0.002), having the phone off during sleep (OR=3.311, p<0.001), and using social media apps (OR=2.797, p=0.005) were linked to an increased risk of sleep disturbancess. Conversely, video-watching (OR=0.349, p=0.007) and moderate music listening (OR=0.220, p=0.004) were associated with a reduced risk of sleep disturbancess. Further analysis indicated that behaviors such as playing games and having the phone off during sleep significantly increased the risk of sleep disturbancess across different genders and academic years (OR>1, p<0.05).

**Conclusion:**

During the COVID-19 pandemic, smartphone-related behaviors such as having the phone off during sleep, playing games before sleep, and using social media apps were identified as risk factors affecting sleep quality. This study provides empirical evidence for developing interventions aimed at improving sleep quality among college students in the context of heightened stress and disrupted routines caused by the pandemic.

## Introduction

In today’s digital-centric society, smartphones have become an integral part of daily life, particularly among college students, the most active demographic of users [1,2]. This trend was even more pronounced during the COVID-19 pandemic when smartphones played a key role in providing education, entertainment, e-commerce, and social connectivity [3]. However, alongside their widespread use, there is a growing concern about smartphone overuse. Recent studies report that 80% of college students use smartphones for more than 4 hours a day, and 44% use them for more than 8 hours a day [4] and indicate that the prevalence of Problematic Smartphone Usage (PSU) [5] among university students varies between 36.5% and 67%, with a mean of 52% [6]. This widespread use of smartphones among college students increases the risk of problematic smartphone use (PSU) and its associated negative consequences, including sleep disturbances, anxiety or depression [2,7].

Problematic Social Media Use (PSMU), a manifestation of improper smartphone use, is defined as a lack of regulation in social media usage leading to impaired daily functioning. Its prevalence is particularly noteworthy in low-income countries, reflecting wider socio-economic influences on digital behavior [8]. Fear of Missing Out (FoMO) is identified as a predisposing factor towards excessive internet and smartphone use, with varying impacts on internet usage among different populations and an intensification during the COVID-19 pandemic [9]. Moreover, Problematic Smartphone Use (PSU) has been significantly associated with sleep disturbances. Diverse psychological constructs such as metacognitions, desire thinking, and emotion regulation have been found to influence the relationship between PSU and sleep disturbances [5].

This study aims to investigate the factors leading to excessive smartphone use and analyze their impact on sleep quality. Specifically, the research will test the following hypothesis:

“Increased evening or night-time smartphone use among college students during the COVID-19 pandemic is negatively correlated with sleep quality, which in turn exacerbates anxiety and depression.”

Although numerous studies have explored the relationship between excessive smartphone use and negative outcomes such as decreased sleep quality, anxiety, and depression, these investigations often fail to comprehensively consider the complex impact of changes in behavior patterns during the pandemic on sleep quality. This study aims to address this research gap by providing new insights into the relationship between smartphone usage habits and the sleep quality status of college students during the COVID-19 pandemic, especially during the lockdown periods when the prevalence and impact of such usage patterns may have been amplified [10,11]. We employed a statistical approach to intricately analyze the association between different types of smartphone usage behaviors (such as gaming, reading, and social media use) and sleep disturbances, thereby offering a scientific basis for the development of targeted interventions to improve sleep quality among college students.

## Methods

### Survey participants

From October to December 2021, and subsequently, from March to May 2022, an academic exploration was carried out at a comprehensive undergraduate university in Anhui, China. This study included a diverse range of students from freshmen to juniors, majoring in fields as varied as humanities, engineering, medicine, and science. Utilizing stratified cluster sampling within classrooms, we distributed 512 questionnaires. This research were rewarded with a remarkable response rate of 99.2%,amounting to 508 retrieved questionnaires. This varied sample comprised 286 male and 222 female participants, aged between 16 and 24, with a mean age of 20.34 (±3.32) years, reflecting the broad demographic landscape of the student body. In this research, Participants were not compensated for their participation in this study. The voluntary nature of participation ensures that responses were given without any incentive bias, reflecting genuine behavior and attitudes towards smartphone usage.

The study involving human participants was approved by the Ethics Committee of Anhui University of Science and Technology (YZ-2021–003) and conducted in accordance with the Declaration of Helsinki. Informed consent forms were obtained from all participants and/or their legal guardians. All identifying information, including names and other HIPAA identifiers, has been removed from the manuscript and supplementary materials.

### Survey methods

The research having undergone rigorous and standardized training, executed a systematic questionnaire survey during a period of confinement. Utilizing a two-pronged approach, participants either engaged with an online format of the questionnaire, submitting their responses via email, or completed a physical copy on-site if they were offline. Upon distribution, the research provided comprehensive instructions to ensure the questionnaire was accurately completed and subsequently collected for centralized processing. The questionnaire was designed to gather a wide array of data, including demographic details such as age, gender, academic year, major, behavior patterns related to mobile phone usage (particularly before bedtime), preferred methods of communication, and related data of sleep.

### Evaluation of sleep quality

In assessing the sleep quality of participants, we employed the Pittsburgh Sleep Quality Index (PSQI). The PSQI is a comprehensive measure of sleep quality, encompassing various aspects such as sleep duration, disturbances, latency, and efficiency.

Buysse et al. (1989), in their seminal work on the PSQI, outline its development and validation [12]. Their study established the clinical and clinimetric properties of the PSQI, assessing “good” sleepers (healthy subjects) and “poor” sleepers (patients with depression and sleep disturbance) [12]. In this study, we utilized the 19-item version of the PSQI, as originally developed and validated by Buysse et al. in 1989. This version is widely recognized for its comprehensive assessment of sleep quality and disturbances; and the sleep quality for each participant was assessed over a span of the past 30 days.

The PSQI assesses seven areas: subjective sleep quality, sleep latency, sleep duration, habitual sleep efficiency, sleep disturbances, use of sleeping medication, and daytime dysfunction. Each of these seven components is scored on a scale from 0 to 3, where 0 indicates no difficulty and 3 indicates severe difficulty. The scores for these components are then summed up to create a global PSQI score, with the total possible score ranging from 0 to 21. A higher score indicates poorer sleep quality [12]. Specifically, we used a cut-off score of 7 in group of Chinese people, where scores equal to or less than this value (≤ 7) indicated normal sleep patterns, and scores greater than this value (> 7) suggested potential sleep disturbances [13,14]. The Cronbach’s α for the PSQI was 0.76, indicating high reliability for values above 0.7.

The PSQI is primarily quantitative, it also encompasses several open-ended questions that can be considered as having qualitative characteristics. For instance, it requires respondents to describe aspects such as the time taken to fall asleep and the number of awakenings during the night. Although these questions yield numerical answers, their essence lies in investigating specific sleep behaviors and experiences, which are regarded as part of qualitative information [12]. The application of the PSQI in our study enabled a robust quantitative evaluation of sleep quality while allowing for qualitative insights into the sleep-related experiences of college students during the COVID-19 pandemic.

### Smartphone use survey

The study examined the nocturnal interaction with smartphones over a one-month period, encompassing activities such as gaming, reading, and learning (view online course) before bedtime; the habit of keeping the phone nearby or if switching off smartphone during sleep; the amusement of media content such as Video-watching; using social media app or late-night phone use; and listening to music as a pre-sleep activity. In this research, we think a response in the survey was considered valid if the average pre-sleep smartphone engagement duration exceeded 30 minutes daily and the cut-off value was depended on times of any single activity was performed before bedtime [15], the cut-off score details see Table notes underneath Table 2. The detail of survey form and PSQI score calculation are demonstrated in S1 and S2 Appendices in S1 File.

**Table 1:** Demographic Characteristics, Scores Among Participants and counts (%) for Smartphone-Related behaviors.

Demographic characteristics	Group	N(%)
Grade	Frosh	204(40.2)
	Sophomore	109(21.5)
	Junior	195(38.3)
Gender	Male	286(56.3)
	Female	222(43.7)
Major	Science.	109(34.7)
	Humanities	232(10.2)
	Engineering	111(29.9)
	Medicine,	56(25.2)
Hometown	Town/City	249(49.0)
	Village/Rural Area	259(51.0)
Seep with phone nearby	none	39(7.7)
	others	469(92.3)
Phone off during sleep	none	38(7.5)
	others	470(92.5)
Sleep with music	none	388(76.4)
	others	120(23.6)
Late-night phone use	none	330(65.0)
	others	178(35.0)
Playing games	none	446(87.8)
	others	62(12.2)
Using social media app	none	200(39.4)
	others	308(60.6)
View online course	none	32(6.1)
	others	477(93.9)
Videos- watching	none	336(66.1)
	others	172(33.9)
Reading before sleep	none	332(65.4)
	others	176(34.6)

**Table 2:** Univariable and Multivariable Model Results with Sleep Disturbances.

Characteristics	p value*	OR (95%CI)*	p value**	OR (95%CI)**
Sleep with phone near by	0.012	0.395(0.191–0.816	0.315	0.592(0.213–1.647)
Reading before sleep	<0.001	2.302(1.391–3.809)	0.186	0.578(0.257–1.303)
Video-watching	0.038	0.777(0.164–0.902)	0.007	0.349(0.163–0.749)
Late-night phone use	<0.001	2.846(1.757–4.609)	0.002	2.824(1.445–5.520)
Playing games	<0.001	3.866(2.158–6.934)	<0.001	6.071(2.323–15.865)
Using social media app	<0.001	2.450(1.461–4.236)	0.005	2.797(1.361–5.747)
View online course	0.005	0.335(0.155–0.724)	0.150	0.446(0.149–1.340)
Sleep with music	0.009	0.379(0.183–0.781)	0.004	0.220(0.079–0.612)
Phone off during sleep	<0.001	3.287(2.022–5.342)	<0.001	3.211(1.859–5.545)

Notes: OR: Odds Ratio (compared with the “none” group); 95% CI: 95% Confidence Interval; *p-value: p-value for ULRA; **p-value: p-value for MLRA. Bold values indicate statistical significance. All variables are dichotomous, coded as follows:

PSQI scores: ≤7 = 0, >7 = 1;

Gender: Male = 1, Female = 2;

Grade: Freshman = 1, Sophomore = 2, Junior = 3. In the analysis, PSQI scores were treated as dependent variables, and related factors affecting sleep quality were independent variables.

### Data analysis

The data analysis was conducted using Microsoft Excel for Mac version 15.18 and SPSS version 27.0 software. For the Pittsburgh Sleep Quality Index (PSQI), we combined the scores of the seven components as described by Buysse et al. (1989) to calculate the total score in detail [12]. A total PSQI score greater than 7 was adopted to identify potential sleep disorders, with this threshold determined based on its diagnostic sensitivity and specificity in distinguishing between “good sleepers” and “poor sleepers” [13,14]. Descriptive statistical methods were used to summarize the data. Spearman’s rank correlation coefficient analysis was conducted to assess the correlation between various smartphone usage behaviors and components of the PSQI.

To understand the impact of smartphone use before bedtime on sleep quality, we adopted a logistic regression approach. This method enabled us to explore the relationship between sleep disorders and various smartphone activities. We examined multicollinearity among the predictor variables using the Variance Inflation Factor (VIF). All statistical tests were two-sided, with p < 0.05 considered statistically significant. The odds ratios (OR) and their 95% CI were calculated to quantify the risk associated with each identified factor.

## Results

### Demographic characteristics and general information

A total of 512 questionnaires were collected, with 508 being valid. The average Pittsburgh Sleep Quality Index (PSQI) score was 6.22 ± 1.5, with a minimum score of 1 and a maximum score of 14. There were 82 participants (15.9% of the total) with PSQI scores greater than 7 (indicating poor sleep quality). Among the participants, 286 were male (56.3%) and 222 were female (43.7%). By academic year, there were 204 freshmen (40.3%), 106 sophomores (21.5%), and 195 juniors (38.3%). The age range of the participants was 17–24 years. For details on Smartphone-Related behaviors, see Table 1.

### The impact of different smartphone-related behaviors on sleep quality among college students

Based on the inclusion criteria (α=0.05) and exclusion criteria (β=0.10), a univariate logistic regression analysis was conducted with the presence or absence of sleep disturbances among college students as the dependent variable. Statistically significant differences (P<0.01) were found among groups with different Smartphone-Related behaviors, indicating a association between these behaviors and sleep quality. As Table 2 indicates that the odds ratios (ORs) for the “Sleep with phone nearby,” “Sleep with music,” and “Videos-watching” groups were less than 1, suggesting a negative association between these smartphone-related behaviors (including “sleeping with the phone nearby” and “sleeping while listening to music”) and sleep quality. These behaviors may help improve sleep quality and reduce the probability of sleep disorders. Conversely, the ORs for the “Late-night phone use,” “Playing games,” “Using social media apps,” “View online course,” “Phone off during sleep,” and “Reading before sleep” groups were all greater than 1, indicating a positive association between these smartphone-related behaviors and the occurrence of sleep disturbances. These behaviors are more likely to cause sleep disturbances. The OR values for playing games and turning off the phone during sleep were 3.866 and 3.287, respectively, indicating that these behaviors increase the probability of sleep disorders by more than three times compared to those without such behaviors.When gender and academic year were included as confounding factors in a multivariate logistic regression analysis, the results showed no significant differences in the ORs for the smartphone-related behaviors of “Sleep with phone near by,” “Reading before sleep,” and “View online course” (P>0.05). However, the ORs for “Playing games,” “Phone off during sleep,” “Late-night phone use,” and “Using social media apps” were greater than 1 (P<0.05), identifying them as risk factors for sleep disturbances. Additionally, the interaction effect between gender and academic year was significant (P<0.05) (see S1 Table).

### Analysis of the association between smartphone-related behaviors and sleep quality among college students of different genders

Based on adjusted analysis results, smartphone-related behaviors such as “Sleep with phone nearby,” “Reading before sleep,” and “View online course” (all with P > 0.05) were excluded. Further analysis of smartphone-related behaviors was conducted. First, the data were processed using SPSS version 27.0 (split by gender) to create “female subset” and “male subset.” Logistic regression analysis was then performed, and odds ratios (ORs) with 95% confidence intervals (CIs) were reported (see Table 3).The results showed that, compared to students without gaming behavior, the probability of sleep disturbances significantly increased among both male and female college students who engaged in “Playing games” (OR = 4.075 for males and 9.766 for females). Gaming behavior was identified as a risk factor for sleep disturbances (P < 0.05, OR > 1). Conversely, “Sleep with music” and “Video-watching” behaviors were associated with varying degrees of improvement in sleep quality for both male and female students (OR < 1).

**Table 3:** The Association Between Smartphone-Related Behaviors and Sleep Quality Stratified by Gender.

	Male		Female	
OR(95%CI)	P	OR(95%CI)	P
Sleep with music	0.084(0.027–0.262)	0.000	0.419(0.087–2.017)	0.278
Video-watching	0.649(0.263–1.602)	0.349	0.073(0.015–0.353)	0.001
Late-night phone use	1.380(0.640–2.977)	0.411	4.550(1.805–11.471)	0.001
Playing games	4.075(1.397–12.044)	0.011	9.766(1.864–51.166)	0.007
Using social media app	4.012(1.484–10.850)	0.006	1.028(0.405–2.611)	0.953
Phone off during sleep	3.549(1.759–7.159)	0.000	2.117(1.882–5.078)	0.033

Table Notes: OR, compared with none (none vs. others)

### Analysis of the association between smartphone-related behaviors and sleep quality among college students of different academic years

The dataset was split by “academic year” using SPSS software to create “freshman,” “sophomore,” and “junior” subsets. A multivariate logistic regression analysis was performed on smartphone-related behaviors, and the results are shown in Table 4.

**Table 4:** The Association of Smartphone-Related Behaviors and Sleep Quality Stratified by Group.

	Frosh		Sophomore		Junior	
OR(95%CI)	P	OR(95%CI)	P	OR(95%CI)	P
Sleep with music	0.065(0.015–.273)	0.000	0.256(0.012–0.703)	0.039	0.300(0.063–1.434)	0.131
Video-watching	0.750(0.275–2.048)	0.575	0.123(0.019–0.796)	0.028	0.079(0.010–0.638)	0.017
Late-night phone use	1.162(0.453–2.978)	0.754	5.422(1.334–22.047)	0.018	4.155(1.458–11.845)	0.008
Playing games	3.414(1.012–11.687)	0.045	21.127(3.058–45.938)	0.002	9.247(1.009–84.758)	0.049
Using social media app	1.640(0.468–5.747)	0.439	1.650(0.458–5.944)	0.444	2.973(0.946–9.113)	0.062
Phone off during sleep	4.339(1.875–10.042)	<0.001	2.745(0.799–9.433)	0.109	2.261(1.852–5.998)	0.044

Table Notes: OR, compared with none(none vs. others)

The analysis revealed that “Playing games” and “Phone off during sleep” were significantly associated with sleep quality among freshmen, sophomores, and juniors. Compared to students without such behaviors, the probability of sleep disturbances significantly increased for freshmen, sophomores, and juniors who engaged in “Playing games” (OR = 3.414, 21.127, and 9.247, respectively), identifying it as a risk factor for sleep disturbances (P < 0.05, OR > 1). Similarly, “Phone off during sleep” also increased the likelihood of sleep disturbances among college students (P < 0.05, OR > 1).

The behavior “Using social media apps” showed no significant impact on students across all academic years (P > 0.05). Notably, juniors exhibited significant differences in behaviors such as “Late-night phone use,” “Playing games,” and “Phone off during sleep” (P < 0.05, OR > 1).

### Correlation analysis between psqi subcomponents and smartphone-related behaviors

Spearman’s rank correlation analysis was employed to examine the correlation between the PSQI subcomponents and various Smartphone-Related Behaviors. The results indicated that the Smartphone-Related Behaviors of “Sleep with music “and “Video-watching” exhibited a negative correlation (r<0,P<0.05) with the PSQI subcomponents, while other Smartphone-Related Behaviors demonstrated a positive correlation (r>0,P<0.05) with the PSQI subcomponents. Additionally,The behaviors of ”playing games” and ”using social media apps” were found to have significant effects on ”sleep duration”, ”sleep efficiency”, and ”sleep latency” (see S1 Table).

## Discussion

Sleep disorders have become a prevalent psychological and physiological phenomenon among contemporary college students, significantly affecting their physical and mental health. This issue has garnered considerable attention from researchers, with studies indicating varying degrees of sleep quality problems among students [16,17]. In the modern era, smartphones have become an indispensable part of every college student’s life. Prolonged and excessive smartphone use has been associated with negative impacts on mental health and behavior, particularly a decline in sleep quality [18]. During the COVID-19 pandemic, measures such as lockdowns, home isolation, school closures, and various epidemic control strategies drastically altered college students’ lifestyles and learning patterns, further impacting their physical and mental health [19]. Smartphones, serving as primary tools for information acquisition, academic communication, entertainment, and psychological comfort, saw changes in usage behaviors, including duration and frequency. These changes were evident not only in daily academic activities, such as online courses and reading, but also in social entertainment and psychological regulation, such as social media use, online gaming, listening to music, and watching videos. Additionally, dependency on smartphones, such as whether to turn them off or keep them nearby, also changed.

This study explores the impact of smartphone usage behaviors on sleep quality among college students during the COVID-19 pandemic, revealing the association between smartphone-related behaviors and sleep disorders during public health emergencies and their control periods. The results, detailed in Table 2, demonstrate the statistical association between college students’ smartphone usage behaviors and sleep quality, providing critical insights into the multidimensional effects of digital habits on sleep. Multivariate logistic regression analysis showed no significant differences in the odds ratios (ORs) for smartphone-related behaviors such as “Sleep with phone nearby,” “Reading before sleep,” and “View online course” (P > 0.05). However, behaviors such as “Playing games,” “Phone off during sleep,” “Late-night phone use,” and “Using social media apps” had ORs greater than 1 (P < 0.05), indicating a higher likelihood of sleep disorders, with “Playing games” being particularly impactful (OR = 6.071). Conversely, “Sleep with music” and “Video-watching” showed some potential in alleviating sleep disorders (OR < 1).

During the COVID-19 pandemic, prolonged home isolation, school closures, and social distancing, coupled with information overload, economic pressure, and health concerns, significantly affected college students’ mental health, exacerbating feelings of loneliness, anxiety, fear, depression, and insomnia [20]. The use of and dependency on various smartphone apps—including games, videos, music, information retrieval, family communication, online courses, and research—increased markedly. While smartphones were used to alleviate depression and anxiety and to stay informed about the pandemic, excessive use of “Playing games” and “Using social media apps” was found to degrade sleep quality. Additionally, “Phone off during sleep” intensified feelings of loneliness, fear, and anxiety, further impairing sleep quality.

This study underscores the complex relationship between smartphone usage behaviors and sleep quality during public health crises, highlighting the need for balanced digital habits to mitigate adverse effects on mental health and sleep.

A study by Sun et al. (2022) specifically analyzed the sleep problems of Chinese university students prior to COVID-19 by including 14 studies involving 21,848 Chinese undergraduate and graduate medical students and found that the prevalence of sleep problems was 12.6%, with a slight increase among undergraduates as their academic years advanced [21]. A longitudinal study named “U-Flourish” by King et al. (2023) analyzed data from 9,523 students between 2018 and 2022 and found that during the COVID-19 pandemic, the average SCI-8 (Sleep Condition Indicator) scores decreased overall (with an average annual change of −0.42; P-trend < 0.001), indicating a deterioration in sleep conditions throughout the academic year and an increased likelihood of potential insomnia at university entry, which suggests a significant decline in sleep quality among university students during the COVID-19 pandemic [22]. Lukowski et al. (2022) compared the sleep, health, and academic performance of American undergraduates before (217 participants from February to December 2019) and during (313 participants from November to December 2020) the COVID-19 pandemic, finding that participants during the pandemic reported poorer overall sleep quality, more severe symptoms of insomnia, and greater stress [23]. ÖZTÜRK et al. (2023) highlighted a significant negative association between sleep quality and levels of coronavirus anxiety and smartphone addiction during the pandemic, pointing to a complex interplay between pandemic-induced anxiety and increased dependence on smartphones that negatively impacts sleep quality [24]. Solon Júnior et al. (2021) indicated that the impact of the pandemic on smartphone usage is associated with symptoms of anxiety, depression, stress, tension, confusion, and insomnia—particularly in physically inactive individuals during self-isolation—thereby highlighting significant changes in daily life due to lockdowns and social distancing measures that may exacerbate the psychological effects of increased smartphone use [25]. Our research shows a close association between smartphone behavior and sleep disturbance among college students, but it is necessary to consider that the unique stress environment of the pandemic may have amplified this relationship. Isolation and prolonged stay at home might increase screen time and reliance on smartphones, thereby reflecting an increase in sleep disturbance. Therefore, when assessing the generalizability of these findings to other contexts, the particularity of the pandemic’s impact on smartphone use and sleep quality must be considered.

Several studies have provided insights into the impact of smartphone usage on sleep quality and the potential benefits of face-to-face interactions. Arumugam et al. (2020) found that excessive nighttime smartphone use among college students negatively affects sleep quality and overall well-being—leading to difficulties in concentration, daytime fatigue, and increased anxiety when separated from the phone—and although the study did not directly measure the impact of face-to-face interaction on smartphone usage, it underscored these adverse effects [26]. Additionally, Lee et al. (2023) investigated the impact of COVID-19 and non-face-to-face courses on adolescent sleep satisfaction and found that during the pandemic these courses increased sleep satisfaction, improved psychological states, boosted physical activities and smartphone usage, and reduced smoking and drinking, suggesting that even without face-to-face interactions changes in social interaction modes can significantly affect sleep and behavioral patterns [27]. Another relevant study by van Bindsbergen et al. (2022) explored the use of social robots for sleep hygiene education among children with cancer, and their preliminary findings suggest that using these robots is feasible, provides positive experiences, and improves children’s sleep hygiene two weeks after the educational program [28]. However, further research is required to develop and implement such interventions. While this study did not directly focus on face-to-face interaction or smartphone usage, it highlighted the potential of alternative intervention measures for improving sleep quality.

Although research indicates that smartphone usage can impair sleep quality, the effectiveness of reducing its impact by increasing face-to-face interactions remains uncertain. More empirical research is needed to explore the impact of interpersonal interactions on improving sleep hygiene among university students. Moreover, our study is limited by sample representativeness,bias in self-reported data, inability to determine causality, and the unique impact of the COVID-19 pandemic. Future research should employ diverse samples and longitudinal designs, and utilize objective methods to assess the relationship between smartphone use and sleep quality, to better understand the complex interaction between the two. The detailed insights from this research provide a robust empirical foundation for developing interventions aimed at improving sleep quality among college students. By identifying specific high-risk behaviors, such as gaming and reading on smartphones before bed, the study advocates for targeted behavioral modifications to enhance sleep quality during a pandemic period marked by heightened stress and disrupted routines.

## Supporting information

S1 File S1 Appendix. Smartphone Use Survey Form. S2 **Appendix**. PSQI Component and Total Score Calculation.
(DOCX)

S1 Table Correlation Analysis Between smartphone-related behaviors and PSQI Sub-components. (DOCX)
