# Supplementary material for: Investigation on the association between college students’ smartphone-related behaviors and sleep quality during COVID-19
Source: PLoS One. 2025 Apr 29;20(4):e0321060. doi: 10.1371/journal.pone.0321060 (PMC12040136; doi:10.1371/journal.pone.0321060)
Supplement: S1 File — S2 Appendix. PSQI Component and Total Score Calculation. (DOCX) [file pone.0321060.s001.docx]

**S1 Appendix Smartphone Use Survey Form**

Identifier:

**Smartphone Use Survey Form**

1. Gender: ① Male; ② Female

2. Race: ① Han; ② Other

3. Date of Birth: (Year-Month)

4. Academic Year: ① Freshman; ② Sophomore; ③ Junior; ④ Senior

5. Major Category: ① Arts; ② Science; ③ Medicine; ④ Arts; ⑤ Other

6. The mobile media device you use the most includes: ① Mobile phone; ② iPad/Tablet; Other

7. How long do you play with your mobile phone before going to sleep?

A. 0-0.5 hours B. More than 0.5 hours

8. Does using smart mobile media devices affect your ability to fall asleep?

A. Never B. Yes

9. Where do you usually keep your mobile phone during sleep?

A. Under the pillow B. In the pocket of clothes or backpack C. Away from the head D. By your side

10. Do you turn off your mobile phone during sleep?

A. Off B. On C. Sometimes off, sometimes on

11. Do you use your phone at midnight?

A. Often; B. No

12. Do you often use your mobile phone for chatting before sleep?

A. Never; B. ≤ 4 times per week; C. > 4 times per week; D. Every night before sleep

13. Do you often use your mobile phone to play games before sleep?

A. Never; B. ≤ 4 times per week; C. > 4 times per week; D. Every night before sleep

14. Do you often use your mobile phone to listen to music before sleep?

A. Never; B. ≤ 4 times per week; C. > 4 times per week; D. Every night before sleep

15. Do you often use your mobile phone for reading before sleep?

A. Never; B. ≤ 4 times per week; C. > 4 times per week; D. Every night before sleep

16. Do you often use your mobile phone to watch videos before sleep?

A. Never; B. ≤ 4 times per week; C. > 4 times per week; D. Every night before sleep

**S2 Appendix PSQI Component and Total Score Calculation**

**PSQI Component and Total Score Calculation**

Subjective Sleep Quality

Based on item 6, responses are scored as follows:

- Very poor: 3 points

- Fairly poor: 2 points

- Fairly good: 1 point

- Very good: 0 points

Sleep Latency

For item 2 (time to fall asleep, in minutes):

- ≥ 61 minutes: 3 points

- 31–60 minutes: 2 points

- 16–30 minutes: 1 point

- ≤ 15 minutes: 0 points

For item 5a (frequency of difficulty falling asleep):

- None: 0 points

- Less than once per week: 1 point

- Once or twice per week: 2 points

- Three or more times per week: 3 points

Combined scoring (sum of item 2 and item 5a):

- Total of 5–6: 3 points

- Total of 3–4: 2 points

- Total of 1–2: 1 point

- Total of 0: 0 points

Sleep Duration

Based on item 4 (hours of actual sleep):

- < 5 hours: 3 points

- 5–6 hours: 2 points

- 6–7 hours: 1 point

- ≥ 7 hours: 0 points

Sleep Efficiency

1. Calculate total time in bed: item 3 (getting up time) – item 1 (bedtime).

2. Compute sleep efficiency: Sleep Efficiency = (Sleep Duration / Time in Bed) × 100%

PSQI Efficiency Component (D) is scored as:

- ≥ 85%: 0 points

- 75%–84%: 1 point

- 65%–74%: 2 points

- < 65%: 3 points

Sleep Disturbance

For items 5b to 5j (various sleep-disturbing symptoms), each item is scored as:

- None: 0 points

- Less than once per week: 1 point

- Once or twice per week: 2 points

- Three or more times per week: 3 points

Combined scoring (sum of items 5b to 5j):

- Total of 0: 0 points

- Total of 1–9: 1 point

- Total of 10–18: 2 points

- Total of 19–27: 3 points

Use of Hypnotic Medication

Based on item 7, scored as:

- None: 0 points

- Less than once per week: 1 point

- Once or twice per week: 2 points

- Three or more times per week: 3 points

Daytime Dysfunction

For item 8, responses are scored as follows:

- None: 0 points

- Less than once per week: 1 point

- Once or twice per week: 2 points

- Three or more times per week: 3 points

For item 9, responses are scored as follows:

- Not at all: 0 points

- Occasionally: 1 point

- Sometimes: 2 points

- Frequently: 3 points

Combined scoring (items 8 and 9):

- Sum of 0: Component G = 0 points

- Sum of 1–2: Component G = 1 point

- Sum of 3–4: Component G = 2 points

- Sum of 5–6: Component G = 3 points

S1 Table Correlation Analysis Between smartphone-related behaviors and PSQI Sub-components

| Behavior | Sleep Duration | | Sleep Efficiency | | Sleep Latency | | Sleep Disturbances | | Subjective Sleep Quality | | Daytime Dysfunction | | Use of sedatives | |
| --- | --- | --- | --- | --- | --- | --- | --- | --- | --- | --- | --- | --- | --- | --- |
|  | *r* | *P* | *r* | *P* | *r* | *P* | *r* | *P* | *r* | *P* | *r* | *P* | *r* | *P* |
| Phone off during sleep | 0.159 | <0.001 | 0.108 | 0.016 | 0.102 | 0.022 | -0.008 | 0.866 | -0.028 | 0.526 | 0.101 | 0.023 | 0.047 | 0.290 |
| Videos- watching | 0.029 | 0.0513 | -0.023 | 0.604 | -0.012 | 0.782 | -0.92 | 0.039 | -0.103 | 0.020 | -0.066 | 0.140 | -0.113 | 0.011 |
| Late-night phone use | 0.046 | 0.300 | 0.132 | 0.003 | 0.011 | 0.789 | 0.004 | 0.926 | 0.031 | 0.482 | 0.132 | 0.003 | 0.010 | 0.328 |
| Playing games | 0.082 | 0.036 | 0.151 | <0.001 | 0.082 | 0.044 | 0.041 | 0.363 | 0.017 | 0.710 | 0.143 | <0.001 | 0.048 | 0.282 |
| Using social media app | 0.137 | 0.002 | 0.095 | 0.033 | 0.134 | 0.003 | 0.026 | 0.557 | 0.048 | 0.279 | 0.050 | 0.261 | 0.085 | 0.044 |
| Sleep with music | 0.056 | 0.205 | -0.008 | 0.862 | -0.093 | -0.035 | -0.461 | 0.001 | 0.033 | 0.007 | -0.009 | 0.025 | -0.099 | 0.025 |

Table Notes: *r*: Spearman's *r*; *P: P* (2-tailed)
