# Supplementary material for: Investigation on the association between college students’ smartphone-related behaviors and sleep quality during COVID-19
Source: PLoS One. 2025 Apr 29;20(4):e0321060. doi: 10.1371/journal.pone.0321060 (PMC12040136; doi:10.1371/journal.pone.0321060)
Supplement: S1 Table — (DOCX) [file pone.0321060.s002.docx]

S1 Table. Correlation Analysis Between smartphone-related behaviors and PSQI Sub-components

| Behavior | **Sleep Duration** | | Sleep Efficiency | | Sleep Latency | | **Sleep Disturbances** | | Subjective Sleep Quality | | Daytime Dysfunction | | **Use of** sedatives | |
| --- | --- | --- | --- | --- | --- | --- | --- | --- | --- | --- | --- | --- | --- | --- |
|  | ***r*** | ***P*** | ***r*** | ***P*** | ***r*** | ***P*** | ***r*** | ***P*** | ***r*** | ***P*** | ***r*** | ***P*** | ***r*** | ***P*** |
| Phone off during sleep | 0.159 | <0.001 | 0.108 | 0.016 | 0.102 | 0.022 | -0.008 | 0.866 | -0.028 | 0.526 | 0.101 | 0.023 | 0.047 | 0.290 |
| Videos- watching | 0.029 | 0.0513 | -0.023 | 0.604 | -0.012 | 0.782 | -0.92 | 0.039 | -0.103 | 0.020 | -0.066 | 0.140 | -0.113 | 0.011 |
| Late-night phone use | 0.046 | 0.300 | 0.132 | 0.003 | 0.011 | 0.789 | 0.004 | 0.926 | 0.031 | 0.482 | 0.132 | 0.003 | 0.010 | 0.328 |
| Playing games | 0.082 | 0.036 | 0.151 | <0.001 | 0.082 | 0.044 | 0.041 | 0.363 | 0.017 | 0.710 | 0.143 | <0.001 | 0.048 | 0.282 |
| Using social media app | 0.137 | 0.002 | 0.095 | 0.033 | 0.134 | 0.003 | 0.026 | 0.557 | 0.048 | 0.279 | 0.050 | 0.261 | 0.085 | 0.044 |
| Sleep with music | 0.056 | 0.205 | -0.008 | 0.862 | -0.093 | -0.035 | -0.461 | 0.001 | 0.033 | 0.007 | -0.009 | 0.025 | -0.099 | 0.025 |

Table Notes: r: Spearman's r; P: P (2-tailed)
